# Cataract—Clinical Lecture of Frank Trester Smith, A. M., M. D., Professor of Diseases of the Eye in the Chattanooga Medical College

**Published:** 1894-11

**Authors:** E. B. Clark


### CATARACT.

Clinical Lecture of Frank Trester Smith, A. M., M. D., Professor of Diseases of the Eye in the Chattanooga Medical College.

REPORTED BY E. B. CLARK.

Mr. B. F. G., age 36, presented himself with the following history:

Was shot in the right eye 4 years ago. Immediately became blind, and has been so ever since. Pain in right eye is occasional. These pains are sharp and lancinating in character. Eye is round and full. Pupil is clouded. Lids normal. About one line from edge of cornea is a dark-blue spot, about the size of a squirrel shot, which is located just above the insertion of the internal rectus muscle. Cornea and iris appear normal. There is gray opacity (Cataract) in the pupilary space.

In this case, from the external appearance, the right eye could be operated upon for the removal of cataract, but in all cases of cataract we have to determine whether or not the eye behind the cataract is healthy, for if there is some pathological condition in the posterior part of the eye-ball, it might counteract any good effect of any operation. As we cannot see through the cataract, nor around it, we judge of this by other means, the first and most important of which is the test for perception of light. This test is decisive so far, that if there is no perception of light, an operation would be useless. The test is made by means of a candle in a dark room. The light is held about a foot from the eye and allowed to shine into the eye, and then shaded by the hand. This test must be repeated several times. In case patient has perception of light, we proceed with the other test.

One test is made in the following way: The eye-ball is pressed by the index finger through the upper lid to steady it. The index finger of the other hand is then gently pressed against the upper part of the eye-ball with the lid intervening. We judge of the amount of tension by the resistance.

A third test is that of the reaction of the pupil to light. It is made by allowing a bright light to shine into the eye, and then shading it. If this is otherwise normal, the light shining through the cataract will cause the pupil to contract.

By the first test we find that there is no perception of light. This is enough to decide against the operation. It is a rule that where there is no perception of light, the patient should not be subjected to any expense for the restoration of sight, also where there is no clear cornea. In all other cases your patient should have the benefit of an expert examination.

The second test shows that the tension is minus (–T) in other words it is softer than normal, this in a case of cataract indicates some abnormality which would make the result of an extraction of the cataract doubtful.

The test for the reaction of the pupil requires care in these cases, as it requires a bright light to contract the pupil, and even then it may contract but little. The movement of the edge of the iris in contraction and dilatation are not as great in the aged (where cataracts are generally found) as in the young. It is best to make this test in a dark room with a good lamp, but a rough test can be made by placing in front of a window and shading the eye momentarily with the hand.

In the case before us the examination is unfavorable by all three of the different tests. In addition to this the eye-ball is tender, which generally contra indicates an operation for the restoration of sight. However, as this eye-ball is tender and painful at times, its removal might be indicated for the relief of these symptoms. The above examination determines positively in this case, and will generally decide the question of an operation in a case of cataract. The examination requires no special instruments, and any general practitioner should be able to go through the procedure.

